# Design of Self-Compacting Concrete with Reduced Cement Content by Aggregate Packing Method

**DOI:** 10.3390/ma18010004

**Published:** 2024-12-24

**Authors:** Tomasz Rudnicki

**Affiliations:** Faculty of Civil Engineering and Geodesy, Military University of Technology, 2 Kaliskiego St., 00-908 Warsaw, Poland; tomasz.rudnicki@wat.edu.pl

**Keywords:** SCC, concrete durability, fly ash

## Abstract

This article presents the procedure for designing self-compacting cement concrete characterized by minimal free space and a maximally compacted mineral skeleton. Such a designed mix allows for lower cement consumption and an increased amount of mineral additives. The paper presents a broad analysis of the influence of different aggregate proportions (36 recipes) and verification of the properties of the concrete mix using CEM I 42.5 R cements and fly ash. As a result of the appropriately compacted mineral skeleton, only 17% free space was obtained, which will allow the amount of cement to be reduced from 500 kg/m^3^ to 350 kg/m^3^ while fully maintaining the properties of the mix and hardened concrete. After 90 days of curing, SCC concrete was characterized by a compressive strength above 68 MPa and a small 2.1% decrease in compressive strength after 100 freeze–thaw cycles.

## 1. Introduction

Self-compacting concrete (SCC) is an innovative type of special concrete. It is characterized by specific rheological properties of a fluid concrete mix due to the precise selection of ingredients and their mutual proportions [1]. These properties constitute the criterion for identifying a self-compacting mix. A mix with a fluid consistency is able to tightly fill the formwork and envelop and fill dense reinforcement while maintaining a uniform composition and minimal porosity, and what is more, it compacts and deaerates under the influence of its own weight [2,3]. The fluid consistency of the mix is achieved thanks to a large amount of binder and the addition of an appropriate superplasticizer based on polycarboxylates and polyethers in an increased amount (2–4%). Their presence allows us to obtain a uniform structure, enabling the efficient use of cement and additives. Low porosity is achieved by reducing the amount of water added to the concrete and the presence of fine-grained mineral additives (fine fractions tightly fill the air gaps between cement grains). Formwork filling occurs without segregation of components and bleeding (leakage of water from the mixture). After laying, it self-levels, which results in an even and smooth surface. In addition, the process of mechanical vibration and noise, which is a negative factor for health, is eliminated. The lack of mechanical compaction results in a reduction in the thickness of the contact zone, which is usually characterized by much greater porosity than deeper layers of grout [4]. Self-compacting concrete is characterized by a reduced number of air pores by up to 25%. The phenomenon of spontaneous deaeration of the mixture is also considered to be one of the basic rheological properties. The need for layered dosing of the mixture disappears, which, together with the high efficiency of self-compacting concrete, results in a reduction in work time [5] and a reduction in labor input by up to 30%. This concrete undoubtedly improves the technological process, because the mixture does not change its consistency during transport and laying, for up to 3 h. It becomes possible to concrete structures with complex, atypical shapes and high-quality surfaces. The structure of the concrete is homogeneous, which results from the continuous process of laying the mixture and eliminating differences in the degree of compaction of individual layers. The exceptional durability and strength of hardened concrete means that self-compacting concretes are usually also high-quality concretes. When it comes to the technical parameters of SCC concrete, a certain analogy can be seen to ordinary concretes. The main, leading value defining the suitability of concrete for a specific application is compressive strength. It influences other technical features, which in the case of compatibility of the compressive strength of these two types of concrete are also similar. The strength range of self-compacting concretes oscillates around 30–100 MPa, while obtaining concretes of lower classes is often problematic. It turns out that, for concretes of classes lower than C30/37, hardened self-compacting concrete after 28 days is characterized by strength 40–80% higher than originally assumed. This is undoubtedly influenced by the high cement content and the presence of mineral additives, sealing the structure of concrete, affecting the increase in strength and durability. One feature that highlights ambiguities is the resistance of concrete to creep. On the one hand, a larger amount of grout contributes to the increase in creep, and on the other hand, lower porosity limits this phenomenon and concrete is more durable. This issue still requires numerous studies and analyses, as the obtained results indicate that the increase in creep is closely related to the high cement content in the mixture. The situation is similar with shrinkage; it was found that the standard procedures have absolutely no practical application in the case of SCC concretes. A larger amount of grout promotes the phenomenon of shrinkage, while a tight structure and a low w/c ratio limit it [3,4]. Numerous studies show that the issue of adhesion of self-compacting concrete to reinforcement is also more favorable than in the case of ordinary concretes [1,2,3,4,5]. This is mainly due to the homogeneous structure of the self-compacting mixture and the lack of differences in the degree of its compaction [4,5,6]. Lower porosity in the contact zone of the grout with the reinforcement causes tight and precise covering of the bars, for both ordinary and prestressing tendons. Greater strength, uniformity, and tightness of concrete does not always have a 100% positive connotation—in the event of fire, it causes more spalling than in the case of ordinary concrete, and additionally, flaking of the surface layer. One method may be the use of propylene fibers, which, by melting in fire conditions, increase the number of pores in the concrete and loosen its structure, increasing permeability and allowing water vapor to escape. When it comes to greater durability of concrete, it results mainly from the tight structure and low content of micropores [7,8]. In this matter, the issue of the appropriate degree of aeration of the mixture becomes important, which is influenced by both air-entraining admixtures and superplasticizer. This requires strict control of their effect on the properties of the mixture with simultaneous verification of the effects of their action and mutual cooperation. The small number of air pores in SCC concretes and the presence of mineral additives, together with a low w/c ratio, additionally seal and strengthen the microstructure. The phenomenon of absorption and diffusion of gases and liquids occurs much slower in SCC concrete. The carbonation process, which deprives concrete of protective properties towards steel and is initiated by the presence of carbon dioxide, is not more aggressive than in the case of ordinary concretes due to the low value of the diffusion coefficient. Reduced diffusion of chloride ions ensures greater durability and protection of reinforcing steel. The main research objective presented in the article is to present the procedure for designing self-compacting concretes by properly selecting the proportions of aggregates (sand and gravel) in order to obtain the minimum free space (vacuum) [2,5]. Concrete designed in this way will be characterized by a minimum amount of cement and water needed to fill the free spaces. The excess binder necessary for the flow of the concrete mix was obtained by increasing the amount of mineral additives, such as limestone flour or fly ash [7]. A new element in the context of design methods is the presented simplified procedure for designing a mineral skeleton characterized by minimal free space. Additionally, a blocking factor for the mineral aggregate mix was introduced into the calculations [8,9,10].

## 2. Materials and Methods

The research work used:

- CEM I 42.5 R cement in accordance with PN-EN 197-1 [11];
- Natural aggregate of fractions 2/8 mm and 8/16 mm, with a density of 2.65 g/cm^3^ and water absorption of 1.60% and natural sand of fraction 0/2 density of 2.65 g/cm^3^;
- In order to increase the viscosity of the concrete mix and reduce the amount of cement, a mineral additive in the form of fly ash with a density of 2.50 g/cm^3^ was used;
- A third-generation high-range/strong water-reducing admixture SP and VMA viscosity regulators complying with NP EN 934-1 [12] and PN-EN 934-2 [13] (amodified polycarboxylic high-range water-reducing admixture in liquid form with a density of 1.02 to 1.07 g/cm^3^)—tap water in accordance with PN-EN 1008 [14].

### 2.1. Methods

The concept behind the method of blocking aggregate in concrete is as follows. The appropriate selection of fractions and determination of the proportions of coarse aggregate and sand is the key to designing a tight crushed pile [15,16,17]. Such a designed mineral mixture provides the desired consistency and workability, while the resulting minimum free space allows the amount of cement to be limited to the minimum amount necessary to obtain compressive strength. From the experience of the authors of the blocking method [6,15,18], coarse aggregates should be avoided because they are characterized by a larger specific surface area compared to natural gravel aggregates. Excessive filling of the mixture with grout, including both filling empty spaces and surrounding the aggregate surface with grout, can result in increased water demand.

Elimination of the phenomenon of blocking coarse aggregate grains between reinforcing bars and ensuring their correct encapsulation is only possible in the case of correct encapsulation of grains with grout while maintaining appropriate distances between them, which translates into determining the optimal content in the mixture. Van B.K. and S. Tangtermsirikul (Bangkok 1995) [18], in their research on the blocking criterion, considered the influence of both the shape and the size of the grains on the blocking coefficient, taking into consideration natural river aggregate of 0–8 mm and crushed limestone of 8–16 mm. Based on the graph, a formula was developed to determine the possibility of aggregate grains getting blocked in the mixture:

(1) $$\sum_{i=1}^{n}\left(\frac{n_{ai}}{n_{abi}}\right)=\sum_{i=1}^{n}\left[\frac{\left(\frac{V_{ai}}{V_{t}}\right)}{\left(\frac{V_{abi}}{V_{t}}\right)}\right]=\sum_{i=1}^{n}\left(\frac{V_{ai}}{V_{abi}}\right)=1$$

where:

*V_ai_*—volume of a given aggregate fraction;
*V_abi_*—blocking index for a given aggregate fraction (value ≤ 1).

Moreover, the use of crushed aggregates is an important issue. They are characterized by a larger specific surface area and, at the same time, a larger friction surface, which requires the use of a larger content of cement paste [19,20]. Assuming a constant amount of cement in the case of natural and crushed aggregate, increasing the amount of paste involves the need to use a large amount of filler (in extreme cases, its amount in concrete on crushed aggregate can be over 230 kg/m^3^).

The voidness criterion determines the optimal content of coarse aggregate (gravel 8–16 mm), or more precisely, its ratio to the total aggregate in the *Nga* mixture, at which the content of voids will be the smallest. The Nga (content of voids in the coarse aggregate in relation to the total aggregate) coefficient also determines the required amount of cement paste [21,22,23,24,25]. The correct design of the mixture composition is realized in the case of the amount of paste greater than the volume of voids in the crushed pile—only then will we obtain the required consistency of the mixture due to fluidity, and consequently the free filling of the formwork with the mixture. Based on analyses and experience, correlations were developed between the minimum required amount of paste in relation to the share of coarse aggregate content. They show that the smallest value of the required amount of paste (in L/m^3^) can be used in the case of 40–60% coarse aggregate content. The most unfavorable cases occur with a complete lack of 8–16 mm gravel (at the same time, obtaining a self-compacting mixture without the presence of this fraction is often impossible) or with excessive overfilling of the crushed pile with coarse aggregate (when the Nga ratio changes by +/− 0.7, even to a value of 1). The void criterion is usually of secondary importance, but it plays an important role in concreting with a narrow stream, in the case of, for example, building walls.

### 2.2. Functional Design Stages

When analyzing the mechanism of self-compacting concrete, it can be assumed, in accordance with the assumptions of the physicochemical mechanics of dispersion systems, that it is a dispersion system consisting of a dispersed phase (mineral skeleton above 0.25 mm) and a liquid phase (water, cement, filler, superplasticizer, and fine sand < 0.25 mm) [9,15]. The assumptions of the functional method for designing self-compacting concrete are based on Professor Paszkowski’s method, the so-called double-envelopment method, i.e., it was assumed that the coarse aggregate grains are covered with a layer of cement mortar, while the sand grains are covered with cement paste. It was assumed that the functional method consists of 4 stages, each of which is responsible for the selection of a different component, and the final verification of properties takes place at the stage of trial mixing [9]. The stages of the functional method are presented in Table 1:

### 2.3. Designing the Mineral Composition of Concrete

The first stage of design consisted of selecting the components of the concrete mix in order to meet the requirements included in the technical design in terms of compressive strength and exposure class. In the second stage, an analysis was carried out in terms of determining the proportions of mineral aggregate fractions 0/2, 2/8, and 8/16 using the blocking method in order to obtain a mineral mix characterized by minimal free space, i.e., the best packing. The proportions of the 36 analyzed mixes are included in Table 2:

The third stage of design consists of calculating the specific surface area of the aggregate pile and assuming the thickness of the aggregate grain cover in order to separate the grains by the film distance, *b*. The aim of this stage is to determine the minimum amount of grout that will allow the concrete mix to flow without blocking the aggregate grains [9]. Calculation of the minimum amount of cement grout, *Z_cmin_* that will allow the concrete to flow based on the determined free space in the mineral mix and the specific surface area of the aggregate pile was performed according to Formula (2):

(2) $$Z_{c_{min}}=Z_{1}+Z_{2}$$

The minimum amount of grout filling the free space between aggregate grains is calculated using Formula (3) and the minimum amount of grout causing the aggregate grains to be separated by a distance, *b*, is calculated using Formula (4) and is designated $Z_{2}$:

(3) $$Z_{1}=\frac{P_{min}\cdot \;\rho_{wz}\;}{\rho_{0\;max}}$$

where *Z*_1_ is the minimum amount of grout filling the free space in the mineral mixture, *g_z_*/*g_k_*; $P_{min}$—is the minimum free space in the mixture determined experimentally, %; and $\rho_{wz}$ is the density of the cement paste, g/cm^3^.

(4) $$Z_{2}=P_{w\;}\cdot b\cdot \;\rho_{wz}$$

where *Z*_2_ is the minimum amount of grout causing overfilling of free spaces in the mineral mixture, *g_z_*/*g_k_*; *P_w_* is the specific aggregate surface area, cm^2^/g; and *b* is the thickness of the film on the aggregate, cm.

Finally, the requirement for the minimum amount of cement paste [9] enabling the concrete to flow is determined according to Formula (5):

(5) $$Z_{cmin}=\left(\frac{P_{min}\cdot \;\rho_{wz}\;}{\rho_{0\;max}}+P_{w\;}\cdot b\cdot \;\rho_{wz}\right)$$

The minimum amount of cement paste, determined according to the assumptions of this method, should allow the desired fluidity of the designed concrete. The final amount of paste (liquid phase) was determined using Formula (6):

(6) $$Z=Z_{cmin\;}\cdot K$$

where Z is the final amount of grout in the concrete mix, kg/m^3^, and K is the amount of fine and coarse aggregate, kg/m^3^.

According to Formula (7), for absolute volume:

(7) $$\frac{Z}{\rho_{wz}}+\frac{K}{\rho_{k}}=1000$$

where $\rho_{k}$ is the aggregate density, g/cm^3^.

The final amount of aggregate in the concrete mix can be calculated using Formula (8):

(8) $$K=\frac{1000}{\frac{Z_{cmin}}{\rho_{wz}}+\frac{1}{\rho_{k}}}$$

In order to check the correctness of the calculations of all the components of the concrete mix, the so-called absolute volume equation (or tightness condition) should be used, which has the following form [26]:

(9) $$\frac{c}{\rho_{c}}+\frac{k}{\rho_{k}}+\frac{p}{\rho_{p}}+\frac{d}{\rho_{d}}+\frac{w}{\rho_{w}}+\frac{m}{\rho_{m}}=1000$$

where *c* is the amount of cement, kg/m^3^; *k* is the amount of coarse aggregate, kg/m^3^; *p* is the amount of sand, kg/m^3^; *d* is the amount of the chemical admixture (the superplasticizer), kg/m^3^; *w* is the amount of water, kg/m^3^; $\rho_{c}$ is the density of the cement, kg/dm^3^; $\rho_{k}$ is the density of the aggregate, kg/dm^3^; $\rho_{p}$ is the density of the sand, kg/dm^3^; $\rho_{d}$ is the density of the chemical admixture (superplasticizer), kg/dm^3^; $\rho_{w}$ is the density of water, kg/dm^3^; *m* is the amount of limestone dust, kg/m^3^; and $\rho_{m}$ is the density of the limestone dust, kg/dm^3^.

The last stage of the design consists of making a trial batch and testing the concrete mix and hardened concrete to verify the SCC properties (L-box, V-funnel, inverted Abrams cone). As part of the work, compressive strength tests and determination of concrete durability were performed.

## 3. Results

In the first stage, analyzing the project requirements, it was determined that the SCC concrete mix should be characterized by strength class C30/37, consistency class SF2, VS1, VF1 and frost resistance F100. In the second stage, a mineral mix was designed using the blocking method and the results are included in Table 1 and Table 2. Further in the text, Table 3 presents sample calculations for one of the regulations, SCC C2. The aim of this stage was to obtain a mix characterized by minimum free space and maximum density. A summary of the obtained results is presented in Table 4.

In the third stage, the specific surface area was calculated for 36 different aggregate mixes with variable proportions of the content of individual fractions, which allows for the analysis of their impact on the remaining parameters in the design. It was assumed that each of the aggregates must be covered with a paste with a film thickness, b, equal to 0.1 mm for aggregates from 2 to 31.5 mm and 0.02 mm for aggregates below 2 mm.

At this stage, the minimum amount of cement paste, *Z_zmin_ Z_cmin_*, was determined, which will allow filling the free spaces, defined as *Z*_1_ and *Z*_2_, which is responsible for moving the aggregate grains apart by a distance, *b*, without blocking the grains during the flow of the concrete mix. A summary of the obtained results of the minimum amount of cement paste is presented in Table 5. The next step in the design is to determine the amount of aggregate using Formula (8) for the total volume of all components and to determine the amount of filler in the form of fly ash.

In order to verify the values of individual components adopted at the design stage, included in Table 6, Table 7 and Table 8, the final design stage was carried out, i.e., preparation of a batch in the laboratory and determination of the properties of the concrete mix. In terms of the concrete mix, the ability of the mix to flow was determined as the degree of fluidity of the concrete mix using the cone flow method. In parallel, the time t_500_ was determined using this method, measured from the moment the cone was lifted until the mixture flowed out to a diameter of 500 mm (mix viscosity). The time of flow of the concrete mix from the V-funnel was also measured [27,28,29]. The measurement of viscosity using the V-funnel method allowed for the assessment of the viscosity and ability of the self-compacting mix to fill the mold. Both tests described above are helpful in confirming the homogeneity of self-compacting concrete for different proportions of aggregate and the amount of ash added. Flowability was also determined, i.e., the ability of the concrete mixture to flow, without losing its homogeneity or blocking, through limited spaces and narrow gaps, such as densely reinforced areas in the L-box device. The obtained results of the properties of self-compacting concrete mixtures are presented in Table 9 and in Table 10 are presented the results of the strength properties and durability of concrete. Below in Table 3 are sample calculations for one of the regulations, SCC C2:

**Table 3:** Recipe ingredients for SCC C2.

Designing Self-Compacting Concrete by the Aggregate Locking Method				
Recipe Composition SCC C2		[%]	[kg/m^3^]	Density [kg/m^3^]
Aggregate				
30%	Gravel 8/16 mm	23.68	576	2.65
40%	Gravel 2/8 mm	31.57	768	2.65
30%	Sand 0/2 mm	23.68	576	2.64
Filer	Fly ash FA	0.00	0	2.66
Cement	CEM I 42.5 R	6.47	350	3.10
Water		2.91	157	1.00
Admixture	ViscoCrete	0.22	5.24	1.05
-		100.0	2431	

**Table 4:** Calculations of aggregate grain size and volume in m^3^.

C = 34 mm									
			Recipe Ingredients in [%]			Volume [L/m^3^]			Sum:
Sieve [mm]	Daf	C/Daf	Sand 0/2	Gravel 2/8	Gravel 8/16	Sand 0/2	Gravel 2/8	Gravel 8/16	
31.500	-	-	0.00	0.00	0.00	0.00	0.00	0.00	0.00
16.000	27.625	1.231	0.00	0.00	0.00	0.00	0.00	0.00	0.00
8.000	14.000	2.429	0.00	1.00	71.00	0.00	2.90	154.23	157.13
4.000	7.000	4.857	0.00	43.00	26.00	0.00	124.55	56.48	181.03
2.000	3.500	9.714	0.00	46.50	3.00	0.00	134.68	6.52	141.20
1.000	1.750	19.429	9.00	9.50	0.00	19.62	27.55	0.00	47.14
0.500	0.875	38.857	36.00	0.00	0.00	78.50	0.00	0.00	78.50
0.250	0.438	77.714	24.00	0.00	0.00	52.33	0.00	0.00	52.33
0.125	0.219	155.429	23.50	0.00	0.00	51.24	0.00	0.00	51.24
0.075	0.113	302.222	2.50	0.00	0.00	5.24	0.00	0.00	5.45
0.000	0.056	604.444	5.00	0.00	0.00	10.90	0.00	0.00	10.90

**Figure 1:**
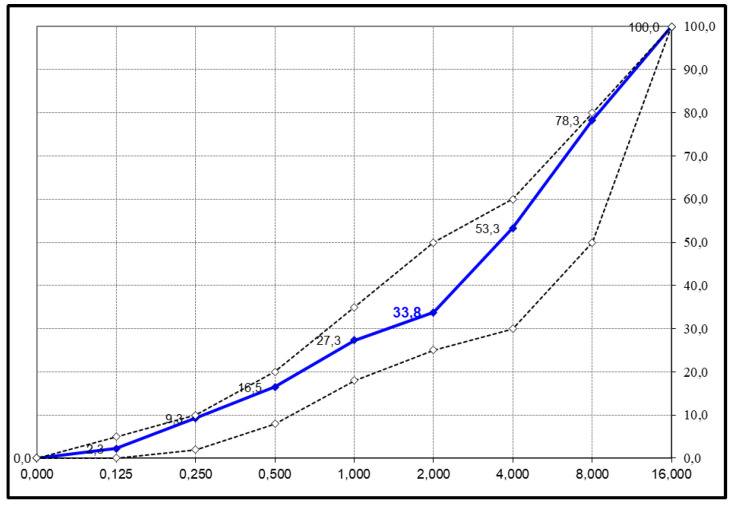
Grain size diagram of the designed mineral mixture.

For the designed aggregate proportions of 30% 8/16 gravel, 40% 2/8 gravel and 30% 0/2 mm sand, the following good grain size distribution curve was obtained, as shown in Figure 1:

**Table 5:** Calculation of the blocking index, BL, for a given aggregate composition, C2.

Blocking for a Given Type of Aggregate						
Sieve	Daf	C/Daf	for Natural Aggregate	for Crushed Aggregate	Sum Volume	Blocking
[mm]			[L/m^3^]	[L/m^3^]	[L/m^3^]	
31.500	-	-	-	-		-
16.000	27.625	1.231	80	60		0.000
8.000	14.000	2.429	508	408		0.326
4.000	7.000	4.857	622	522		0.254
2.000	3.500	9.714	724	670		0.138
1.000	1.750	19.429	840	840		0.050
0.500	0.875	38.857	840	840		0.108
0.250	0.438	77.714	840	840		0.080
0.125	0.219	155.429	840	840		0.087
0.075	0.113	302.222	840	840		0.014
0.000	0.056	604.444	840	840		0.028
						1.088

Values *q*_0*max*_ and *P_min_*, which are necessary to determine the minimum amount of grout, were determined experimentally. The following values of the maximum bulk density, *q*_0*max*_, and free space in the designed mix, *P_min_*, for case C2 were obtained:

$$q_{0\;max}=2.098\;g/{cm}^{3}\;$$

$$P_{min}=20.83\%=0.2083\;g/{cm}^{3}$$

which is why:

$$Z_{1}=\frac{0.2083\cdot 1.81}{2.098}=0.1797\;g_{z}/g_{k}$$

Assuming the density of the cement paste is equal to 1.81 g/cm^3^.

Based on the calculated specific surface area, the demand for grout, *Z*_2_, is determined according to the calculations according to the adopted calculation model:

$$Z_{2}=0.1932\;g_{z}/g_{k}$$

**Table 6:** Determination of the demand for *Z*_2_ grout based on the determined specific surface area and the thickness of the grout film for recipe SCC C2.

SCC C2				
Calculated Specific Surface Area *P_w_*	Wrap Radius *b*	*P_w_* · *b*	Grout Density	Demand for Leaven *Z*_2_
[cm^2^/g]	[cm]	[cm^3^/g]	[g/m^3^]	[*g_z_*/*g_k_*]
0.00	0.01	0.0000	1.81	0.0000
0.00	0.01	0.0000	1.81	0.0000
0.54	0.01	0.0054	1.81	0.0098
1.25	0.01	0.0125	1.81	0.0226
1.95	0.01	0.0195	1.81	0.0353
1.30	0.002	0.0026	1.81	0.0047
4.32	0.002	0.0086	1.81	0.0156
5.76	0.002	0.0115	1.81	0.0209
11.28	0.002	0.0226	1.81	0.0408
2.40	0.002	0.0048	1.81	0.0087
9.60	0.002	0.0192	1.81	0.0348
38.40		0.1067	1.81	0.1932

The calculated amount of grout needed to fill the empty spaces in the mixture is given by *Z*_1_ = 0.1797 $g_{z}/g_{k}$, while the amount of leaven, *Z*_2_, due to the specific surface area and film thickness *b* for this composition is 0.1932 $g_{z}/g_{k}$. The blocking factor for this case is 1.09. In summary, the total slurry requirement (liquid phase) for the SCC C2 recipe is:

$$Z_{cmin}=0.1797+0.1932=0.3729\;\;g_{z}/g_{k}$$

With the above values at our disposal, we calculate the amount of aggregate needed:

$$K=\frac{1000}{\frac{0.3729}{1.81}+\frac{1}{2.6470}}=1712.89\;kg/m^{3}$$

Therefore, the final amount of cement paste (liquid phase) is as follows:

$$Z=0.3729\;\cdot \;1712.89=638.74\;kg/m^{3}\;$$

The amount of grout (cement + water) is known and assumed in the recipe; it is as follows: cement 350 kg and water 157.5 kg, i.e., together, 507.5 kg/m^3^. We have obtained the amount of necessary grout of 638.74 kg/m^3^. This means that the remaining amount (131.24 kg/m^3^) must be supplemented with a filler, e.g., fly ash, so that the concrete can flow freely (is self-compacting) by spreading the aggregate with the grout. The amount of superplasticizer added, 1.5%, to the cement mass is 5.25 kg/m^3^.

Final summary of all grout quantity results, *Z*_1_, *Z*_2_, and *Z_cmin_*, for each of the 36 analyzed compositions are presented in Table 7:

**Table 7:** Calculation of the minimum amount of grout and the aggregate blocking index.

Recipe	Gravel 8/16 [%]	Gravel 2/8 [%]	Sand 0/2 [%]	*q*_0*max*_ [g/cm^3^]	*P_min_* [%]	*q_wz_* [g/cm^3^]	*Z*_1_ [g_z_/g_k_]	*P_w_* [g/cm^2^]	*Z*_2_ [g_z_/g_k_]	*Z_cmin_* [kg/m^3^]	Blocking Indicator
A1	50	50	0	1.648	37.81	1.81	0.4153	6.1	0.0957	0.511	1.218
A2	50	40	10	1.724	34.94	1.81	0.3668	16.5	0.1236	0.490	1.197
A3	50	30	20	1.789	32.49	1.81	0.3287	26.9	0.1515	0.480	1.177
A4	50	20	30	1.855	30.00	1.81	0.2927	37.3	0.1793	0.472	1.157
A5	50	10	40	1.962	25.96	1.81	0.2395	47.8	0.2072	0.447	1.137
A6	50	0	50	2.078	21.58	1.81	0.1880	58.2	0.2351	0.423	1.117
B1	40	50	10	2.172	18.04	1.81	0.1503	17.0	0.1305	0.281	1.169
B2	40	40	20	2.186	17.51	1.81	0.1450	27.4	0.1584	0.303	1.149
B3	40	30	30	2.196	17.13	1.81	0.1412	37.9	0.1863	0.327	1.128
B4	40	20	40	2.199	17.02	1.81	0.1401	48.3	0.2141	0.354	1.108
B5	40	10	50	2.205	16.79	1.81	0.1378	58.7	0.242	0.380	1.088
B6	40	0	60	2.214	16.45	1.81	0.1345	69.2	0.2699	0.404	1.068
C1	30	50	20	2.065	22.08	1.81	0.1935	28.0	0.1653	0.359	1.12
C2	30	40	30	2.098	20.83	1.81	0.1797	38.4	0.1932	0.373	1.1
C3	30	30	40	2.135	19.43	1.81	0.1647	48.8	0.2211	0.386	1.08
C4	30	20	50	2.186	17.51	1.81	0.1450	59.3	0.2489	0.394	1.059
C5	30	10	60	2.199	17.02	1.81	0.1401	69.7	0.2768	0.417	1.039
C6	30	0	70	2.211	16.57	1.81	0.1356	80.1	0.3047	0.440	1.019
D1	20	60	20	2.096	20.91	1.81	0.1806	28.5	0.1723	0.353	1.091
D2	20	50	30	2.119	20.04	1.81	0.1712	38.9	0.2001	0.371	1.071
D3	20	40	40	2.127	19.74	1.81	0.1680	49.4	0.228	0.396	1.051
D4	20	30	50	2.145	19.06	1.81	0.1608	59.8	0.2559	0.417	1.031
D5	20	20	60	2.167	18.23	1.81	0.1523	70.2	0.2837	0.436	1.011
D6	20	10	70	2.185	17.55	1.81	0.1454	80.6	0.3116	0.457	0.991
E1	10	60	30	2.115	20.19	1.81	0.1728	39.5	0.2071	0.380	1.043
E2	10	50	40	2.128	19.70	1.81	0.1676	49.9	0.2349	0.402	1.022
E3	10	40	50	2.142	19.17	1.81	0.1620	60.3	0.2628	0.425	1.002
E4	10	30	60	2.158	18.57	1.81	0.1558	70.8	0.2907	0.446	0.982
E5	10	20	70	2.162	18.42	1.81	0.1542	81.2	0.3185	0.473	0.962
E6	10	10	80	2.181	17.70	1.81	0.1469	91.6	0.3464	0.493	0.942
F1	0	70	30	2.189	17.40	1.81	0.1439	40.0	0.2214	0.358	1.014
F2	0	60	40	2.207	16.72	1.81	0.1371	50.4	0.2419	0.379	0.994
F3	0	50	50	2.244	15.32	1.81	0.1236	60.9	0.2697	0.393	0.974
F4	0	40	60	2.265	14.53	1.81	0.1161	71.3	0.2976	0.414	0.954
F5	0	30	70	2.279	14.00	1.81	0.1112	81.7	0.3255	0.437	0.933
F6	0	20	80	2.286	13.74	1.81	0.1088	92.1	0.3533	0.462	0.913

Knowing the minimum amount of grout, *Z_cmin_*, the remaining components of the concrete mix can be determined from Formulas (5)–(8). The final compositions of the recipes, together with the determination of the minimum amount of fly ash (FA) [29], are presented in Table 8. The calculation of the amount of FA to meet the demand for the Z paste was made assuming that we have a constant amount of cement, C 350 kg/m^3^ and w/c 0.45.

**Table 8:** Composition calculations for the analyzed recipes differing in the amount of individual aggregate fractions and the demand for fly ash FA.

Recipe	Gravel 8/16 [%]	Gravel 2/8 [%]	Sand 0/2 [%]	*P_min_* [%]	*P_w_* [g/cm^2^]	Blocking Indicator	Aggregate [kg/m^3^]	Cement Grout [kg/m^3^]	Cement [kg/m^3^]	Water [kg/m^3^]	FA [kg/m^3^]
A1	50	50	0	37.81	6.1	1.218	1514	773.8	350	157.5	266.3
A2	50	40	10	34.94	16.5	1.197	1541	755.6	350	157.5	248.1
A3	50	30	20	32.49	26.9	1.177	1554	746.4	350	157.5	238.9
A4	50	20	30	30.00	37.3	1.157	1565	738.9	350	157.5	231.4
A5	50	10	40	25.96	47.8	1.137	1600	714.9	350	157.5	207.4
A6	50	0	50	21.58	58.2	1.117	1634	691.5	350	157.5	184.0
B1	40	50	10	18.04	17.0	1.169	1875	526.7	350	157.5	19.2
B2	40	40	20	17.51	27.4	1.149	1833	556.0	350	157.5	48.5
B3	40	30	30	17.13	37.9	1.128	1789	585.8	350	157.5	78.3
B4	40	20	40	17.02	48.3	1.108	1743	617.3	350	157.5	109.8
B5	40	10	50	16.79	58.7	1.088	1701	646.0	350	157.5	138.5
B6	40	0	60	16.45	69.2	1.068	1663	672.3	350	157.5	164.8
C1	30	50	20	22.08	28.0	1.12	1735	622.6	350	157.5	115.1
C2	30	40	30	20.83	38.4	1.1	1712	638.4	350	157.5	130.9
C3	30	30	40	19.43	48.8	1.08	1691	652.6	350	157.5	145.1
C4	30	20	50	17.51	59.3	1.059	1679	661.2	350	157.5	153.7
C5	30	10	60	17.02	69.7	1.039	1644	685.2	350	157.5	177.7
C6	30	0	70	16.57	80.1	1.019	1609	708.7	350	157.5	201.2
D1	20	60	20	20.91	28.5	1.091	1745	615.8	350	157.5	108.3
D2	20	50	30	20.04	38.9	1.071	1715	636.6	350	157.5	129.1
D3	20	40	40	19.74	49.4	1.051	1675	663.5	350	157.5	156.0
D4	20	30	50	19.06	59.8	1.031	1644	685.1	350	157.5	177.6
D5	20	20	60	18.23	70.2	1.011	1616	704.4	350	157.5	196.9
D6	20	10	70	17.55	80.6	0.991	1586	724.7	350	157.5	217.2
E1	10	60	30	20.19	39.5	1.043	1701	646.1	350	157.5	138.6
E2	10	50	40	19.70	49.9	1.022	1665	670.3	350	157.5	162.8
E3	10	40	50	19.17	60.3	1.002	1632	693.2	350	157.5	185.7
E4	10	30	60	18.57	70.8	0.982	1601	714.6	350	157.5	207.1
E5	10	20	70	18.42	81.2	0.962	1564	739.5	350	157.5	232.0
E6	10	10	80	17.70	91.6	0.942	1537	758.2	350	157.5	250.7
F1	0	70	30	17.40	40.0	1.014	1737	621.5	350	157.5	114.0
F2	0	60	40	16.72	50.4	0.994	1702	645.2	350	157.5	137.7
F3	0	50	50	15.32	60.9	0.974	1680	660.6	350	157.5	153.1
F4	0	40	60	14.53	71.3	0.954	1648	682.0	350	157.5	174.5
F5	0	30	70	14.00	81.7	0.933	1615	705.1	350	157.5	197.6
F6	0	20	80	13.74	92.1	0.913	1579	729.6	350	157.5	222.1

The last stage of the design is the experimental verification of the properties of the concrete mix and the strength and durability characteristics of the cement concrete. Analyzing the results of the free space size, blocking index, and specific surface area of the designed aggregate and the resulting demand for the amount of cement paste, the following formula compositions were selected for further tests: B2, B3, B4, B5, C2, C3, C4, C5, D3, and D4. During the preparation of laboratory batches, the properties of the concrete mix were determined in terms of the ability of the mix to flow. The fluidity of the concrete mix was determined according to the cone flow method, in addition to the flow time, t_500_, measured from the moment of lifting the cone to the moment of the mix flowing to a diameter of 500 mm [29,30,31,32,33]. The time of the concrete mix flowing out of the V-funnel was also measured, as well as the ability of the concrete mix to flow without losing its homogeneity or blocking in the L-box device. The obtained results of the properties of self-compacting concrete mixes are presented in Table 9.

**Table 9:** Properties of the analyzed self-compacting concrete mixtures.

Recipe	Gravel 8/16 [%]	Gravel 2/8 [%]	Sand 0/2 [%]	Cement [kg/m^3^]	Water [kg/m^3^]	FA [kg/m^3^]	SP [kg/m^3^]	SF [mm]	VS t_500_ [s]	VF [s]	L-Box >0.80
B2	40	40	20	350	157.5	48.5	4.90	490	4.4	12.1	0.77
B3	40	30	30	350	157.5	78.3	5.25	540	4.0	9.3	0.79
B4	40	20	40	350	157.5	109.8	5.25	630	3.3	10.1	0.81
B5	40	10	50	350	157.5	138.5	4.90	640	3.1	8.4	0.84
C2	30	40	30	350	157.5	130.9	5.60	650	2.6	7.5	0.83
C3	30	30	40	350	157.5	145.1	5.60	670	1.8	6.6	0.85
C4	30	20	50	350	157.5	153.7	5.95	710	1.4	6.9	0.86
C5	30	10	60	350	157.5	177.7	5.60	730	1.5	5.9	0.87
D3	20	40	40	350	157.5	156.0	5.60	750	1.6	5.5	0.86
D4	20	30	50	350	157.5	177.6	5,25	730	1.3	5.7	0.88

**Figure 2:**
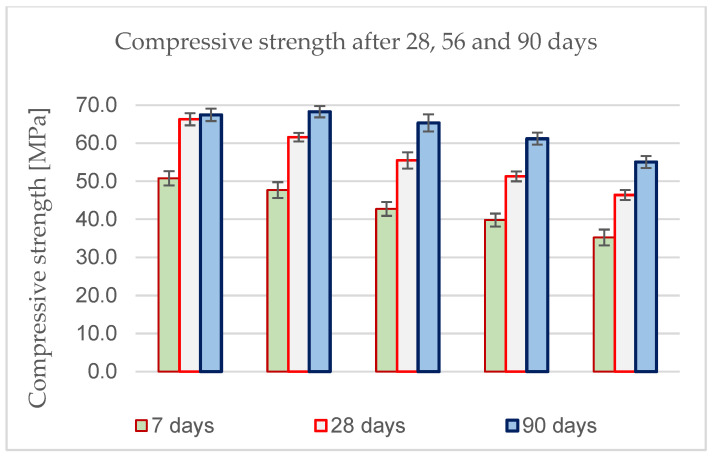
Compressive strength results after 7, 28, and 90 days.

The last stage of experimental verification of the design of SCC concrete with reduced cement content was a series of strength tests and durability tests for the selected recipes. The results of compressive strength performed after 7, 28, and 90 days are presented in Figure 2 below:

The full results of determining the compressive strength, frost resistance, F100, and the air entrainment structure of concrete are presented in Table 10:

**Table 10:** Determination of compressive strength, frost resistance, F100, and air entrainment structure of SCC concrete.

Recipe	Gravel 8/16	Gravel 2/8	Sand 0/2	Cement	Water	FA	Compressive Strength			F100	*A*_300_	*L*
	[%]	[%]	[%]	[kg/m^3^]	[kg/m^3^]	[kg/m^3^]	[MPa]	[MPa]	[MPa]	[MPa]	[%]	[mm]
							After 7 Days	After 28 Days	After 90 Days			
B2	40	40	20	350	157.5	48.5	40.5	51.4	60.2	−31	1.96	0.27
B3	40	30	30	350	157.5	78.3	42.1	57.2	63.5	−1.9	2.05	0.24
B4	40	20	40	350	157.5	109.8	40.6	60.3	68.3	−3.8	1.91	0.22
B5	40	10	50	350	157.5	138.5	43.3	55.7	64.2	−7.7	2.56	0.17
C2	30	40	30	350	157.5	130.9	47.7	61.6	68.3	0.8	2.51	0.20
C3	30	30	40	350	157.5	145.1	50.8	60.3	67.4	−2.1	2.90	0.15
C4	30	20	50	350	157.5	153.7	48.4	61.4	69.8	0.9	3.31	0.09
C5	30	10	60	350	157.5	177.7	41.8	63.7	72.5	−4.1	2.29	0.16
D3	20	40	40	350	157.5	156.0	44.5	58.9	68.6	−3.0	2.36	0.21
D4	20	30	50	350	157.5	177.6	42.5	56.4	66.7	−8.9	1.88	0.26

## 4. Discussion

The presented research work presented a procedure for designing self-compacting concretes using a reduced amount of cement by designing a composition of the aggregate mixture that will be characterized by the smallest vacuum. Although the design of self-compacting concrete is a much more complicated process and requires increased control and knowledge of innovative concretes, it allows obtaining a mixture without the need for vibration. Hardened SCC concrete surpasses ordinary concretes in its properties and technical parameters; it provides new possibilities and a spectrum of applications and could revolutionize the approach to design and execution in construction. Despite the lack of unambiguous design methods in Poland, it is worth using the available achievements of European countries, where self-compacting concrete technology is widely used. The emerging methods are based on recommendations regarding SCC and are still being developed; an example is the blocking method, according to which the composition of the concrete mixture was designed. For 36 designed mineral aggregate formulas, the grain size distribution curve, specific surface area, minimum value of the demand for grout, and the blocking index of the aggregate composition were calculated. A large number of the considered mixture compositions allowed for a broad analysis of the obtained results and the drawing of conclusions. It should be stated that the obtained results confirmed compliance with the theoretical assumptions regarding the composition of SCC mixtures. From among the analyzed cases, several compositions were selected that were optimal in terms of free spaces and the blocking index. Of course, this is not the only correct composition of the mixture, but when selecting it all parameters were taken into account and an attempt was made to select the most advantageous variant in terms of technology and economy. Further experimental research and continuous expansion of knowledge in the field of SCC concretes is extremely important and will certainly contribute in the future to the free use of self-compacting concrete technology, which is increasingly commonly used in special and architectural concretes in Poland. In further studies, the research will be extended to include studies involving crushed aggregates (basalt, granite) and other additives, such as blast furnace slag or limestone flour.

## 5. Conclusions

Based on the calculations, tests, and analyses performed for 36 different formulations, the following conclusions can be drawn:

1. The increase in the specific surface area of the aggregate causes an increase in the demand for cement paste, which significantly affects the increase in the amount of cement.
2. The greatest demand for paste is characteristic for mixtures with a high sand fraction content, above 40%, which confirms the theoretical assumption of the blocking method, while mineral mixtures with the largest specific surface area are characterized by the lowest blocking index.
3. In most cases, the grain size curve did not fit into the limit curves recommended for road and bridge concretes; therefore, new limit curves should be determined for SCC. The grain size curve for cases from group F did not fit into the limit curves, which indicates that obtaining self-compacting concrete without a gravel fraction of 8/16 is very difficult and does not guarantee obtaining the assumed strength parameters,
4. The analyzed recipes, in which the share of coarse aggregate was 60–70% and sand was 30–40%, were characterized by the lowest demand for grout and were within the limit curves. This was confirmed in the SCC tests.
5. After analyzing 36 different mineral compositions of self-compacting concretes for further analysis, the smallest free space and the lowest blocking index were found in formulas from groups B2–B4, C2–C5, and D3–D4.
6. The use of this method allows for a significant reduction in the content of voids and the amount of cement in the SCC mixture without a significant decrease in the flow properties and durability characteristics of concrete over time.

## Figures and Tables

**Table 1:** Design stages for SCC [9].

Stage	Description	Stage Objective
Stage I	Determination of w/c, type of cement, type of mineral additives and chemical admixture	Achieving the project requirements contained in the technical specifications
Stage II	Selection of grain size of the mineral mixture by the blocking method	Obtaining a mineral mixture with minimal free space
Stage III	Calculation of the specific surface area of the aggregate pile and assumption of the thickness of the aggregate grain covering in order to separate the grains by the film distance, *b*	Determining the minimum amount of grout that will allow the concrete mix to flow without blocking the aggregate grains
Stage IV	Preparation of a test batch, testing of the concrete mix and hardened concrete	Verification of SCC properties (L-box, V-funnel, Abrams inverted cone, strength and durability tests)

**Table 2:** Analyzed proportions of the mineral mixture (%).

	**Recipes**	**1**	**2**	**3**	**4**	**5**	**6**
A	Gravel 8/16	50	50	50	50	50	50
	Gravel 2/8	50	40	30	20	10	0
	Sand 0/2	0	10	20	30	40	50
B	Gravel 8/16	40	40	40	40	40	40
	Gravel 2/8	50	40	30	20	10	0
	Sand 0/2	10	20	30	40	50	60
C	Gravel 8/16	30	30	30	30	30	30
	Gravel 2/8	50	40	30	20	10	0
	Sand 0/2	20	30	40	50	60	70
D	Gravel 8/16	20	20	20	20	20	20
	Gravel 2/8	60	50	40	30	20	10
	Sand 0/2	20	30	40	50	60	70
E	Gravel 8/16	10	10	10	10	10	10
	Gravel 2/8	60	50	40	30	20	10
	Sand 0/2	30	40	50	60	70	80
F	Gravel 8/16	0	0	0	0	0	0
	Gravel 2/8	70	60	50	40	30	20
	Sand 0/2	30	40	50	60	70	80

## Data Availability

The original contributions presented in this study are included in the article. Further inquiries can be directed to the corresponding author.
